# Paper-based fluorescence sensor array with functionalized carbon quantum dots for bacterial discrimination using a machine learning algorithm

**DOI:** 10.1007/s00216-024-05262-4

**Published:** 2024-04-17

**Authors:** Fangbin Wang, Minghui Xiao, Jing Qi, Liang Zhu

**Affiliations:** 1 School of Food and Biological Engineering, Hefei University of Technology, Hefei, 230009 China; 2 Department of Chemistry, National University of Singapore, Singapore, 117543 Singapore; 3 Department of Biomedical Engineering, The Hong Kong Polytechnic University, Hong Kong, 999077 China

**Keywords:** Bacterial discrimination, Sensor array, CQDs, Machine learning

## Abstract

The rapid discrimination of bacteria is currently an emerging trend in the fields of food safety, medical detection, and environmental observation. Traditional methods often require lengthy culturing processes, specialized analytical equipment, and bacterial recognition receptors. In response to this need, we have developed a paper-based fluorescence sensor array platform for identifying different bacteria. The sensor array is based on three unique carbon quantum dots (CQDs) as sensing units, each modified with a different antibiotic (polymyxin B, ampicillin, and gentamicin). These antibiotic-modified CQDs can aggregate on the bacterial surface, triggering aggregation-induced fluorescence quenching. The sensor array exhibits varying fluorescent responses to different bacterial species. To achieve low-cost and portable detection, CQDs were formulated into fluorescent ink and used with an inkjet printer to manufacture paper-based sensor arrays. A smartphone was used to collect the responses generated by the bacteria and platform. Diverse machine learning algorithms were utilized to discriminate bacterial types. Our findings showcase the platform's remarkable capability to differentiate among five bacterial strains, within a detection range spanning from 1.0 × 10^3^ CFU/mL to 1.0 × 10^7^ CFU/mL. Its practicality is further validated through the accurate identification of blind bacterial samples. With its cost-effectiveness, ease of fabrication, and high degree of integration, this platform holds significant promise for on-site detection of diverse bacteria.

**Graphical abstract:**

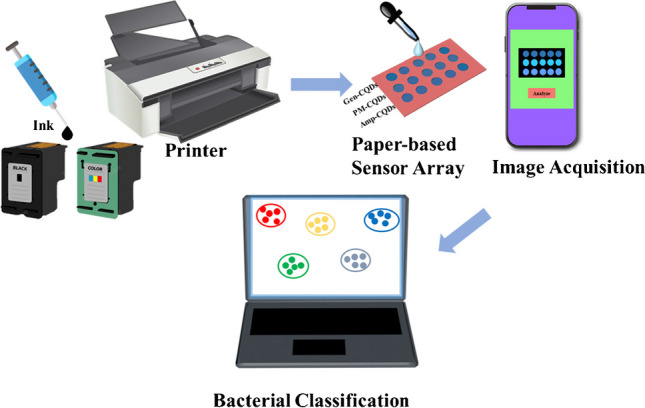

**Supplementary Information:**

The online version contains supplementary material available at 10.1007/s00216-024-05262-4.

## Introduction

The rapid and accurate identification of various bacterial strains is of paramount importance for effective disease control, treatment, and prevention, and for ensuring the safety and quality of food products and the environment [1]. While traditional bacterial recognition methods, including germiculture [2], microscopic observation of bacterial samples [3], flow cytometry [4], and enzyme-linked immunosorbent assays (ELISA) [5], have been established as reliable, these methods are encumbered by limitations such as prolonged culture periods, the need for advanced analytical equipment, the demand for specialized technical expertise, and location-specific restrictions, which impede their broad application.

Fluorescence analysis technology, known for its high sensitivity, specificity, and accuracy, is employed extensively in sensor development [6]. Current research in bacterial detection via fluorescence sensors focuses primarily on the unique interactions between the sensor and bacteria, leading to fluorescence quenching [7]. The recognition elements include antimicrobial peptides [8, 9], antibodies [10], bacteriophages [11, 12], and aptamers [13, 14]. Furthermore, the selection of fluorescent moieties is crucial for sensor sensitivity [15]. Consequently, fluorescent nanomaterials characterized by their high quantum yield, exceptional stability, and prolonged fluorescence lifetime are widely utilized in crafting sensors. Notably, carbon quantum dots (CQDs), a type of zero-dimensional carbon nanomaterial smaller than 10 nm, are valued for their straightforward synthesis, stable fluorescence, and minimal cytotoxicity, making them promising candidates for fluorescent sensing applications [16]. The diminutive size and potent adsorption properties of CQDs facilitate their enhanced accumulation on bacterial surfaces, leading to more distinct fluorescence labeling [17]. CQDs can also be engineered in various colors to detect different bacterial types. Nevertheless, despite the specificity afforded by traditional lock-and-key mechanism sensors, they struggle to detect multiple analytes simultaneously and are susceptible to interference from structurally similar substances [18].

Sensor arrays, characterized by their high-throughput capabilities, serve as efficient tools for the simultaneous detection and identification of diverse analytes [19]. Unlike traditional sensors that demand high specificity, these arrays utilize a variety of nonspecific sensing elements that mimic mammalian taste and olfactory systems [20, 21]. These elements generate a broad range of responses, collectively forming distinct patterns that aid in the recognition of multiple analytes. A key advantage of sensor arrays lies in their ability to address the challenges associated with the development of specific recognition receptors, offering significant benefits such as the recognition of a wide array of targets, robust interference resistance, concurrent detection capabilities, and the ability to differentiate between targets with similar structures or features [22]. Recent research has demonstrated that the integration of machine learning (ML) techniques, including linear discriminant analysis, decision trees, and *k*-nearest neighbors, into sensor arrays can significantly improve the accuracy of pattern recognition in analyte detection [23]. At present, various sensor arrays employing distinct recognition mechanisms have been developed for bacterial detection, leveraging differences in bacterial volatile compounds [24], metabolite [25], and surface electronegativity [26]. Nonetheless, there are certain limitations in the current design of sensor arrays for bacterial differentiation, especially in the fabrication of sensing elements and the portability of platforms. This underscores the urgent need for the development of simple, portable, and rapid sensor arrays for field detection of bacteria. Paper, as a substrate, offers multiple advantages, such as cost-effectiveness, a high surface area-to-volume ratio, and a fibrous network structure conducive to storing reaction reagents [27, 28]. Nowadays, paper-based analytical devices have been successfully combined with fluorescence analysis techniques to detect a variety of analytes [29, 30].

In this study, we developed a straightforward, inkjet-printed paper-based fluorescent sensor array using three types of antibiotics with unique bacterial affinity to synthesize CQDs as sensing units. The chemical composition of bacterial surfaces mainly consists of carbohydrates, proteins, and lipids, with the differences among different bacterial surfaces primarily reflected in the concentrations of these components. Exploiting the characteristics of antibiotics (polymyxin B, ampicillin, and gentamicin) to specifically bind to bacterial surface lipopolysaccharides and proteins [31–34], we prepared three types of antibiotic-modified CQDs as sensing elements. In the presence of bacteria, these antibiotic-modified CQDs can aggregate on the bacterial surface, triggering aggregation-induced fluorescence quenching. The different binding interactions of the three antibiotics with bacterial surfaces result in varying quenching effects of the CQDs. To achieve low-cost and portable detection, CQDs were formulated into fluorescent ink and used with an inkjet printer to manufacture paper-based sensor arrays. Presented with different bacteria, this sensor array exhibits unique fluorescent responses to each. These different responses are captured and analyzed with a smartphone and ML, enabling the identification of distinct patterns for bacterial recognition. This sensor array offers multiple benefits, such as portability, precision, and speed, presenting a new avenue for applications in food safety, medical diagnostics, and environmental monitoring.

## Materials and methods

### Materials

Ammonium citrate dibasic, alkylketene dimer (AKD), glycol, Triton X-100, polymyxin B, ampicillin, and gentamicin were purchased from Adamas Reagent Co., Ltd. (Shanghai, China). All the reagents were of analytical grade and were used directly. Filter paper was purchased from Hangzhou Double Circle Filter Paper Co., Ltd. (Hangzhou, China). Ultrapure water (18.2 MΩ cm) was used in all aqueous solutions.

### Instruments

An Eppendorf BioSpectrometer Basic was used to measure the optical density (OD) value of the bacteria. An Agilent Cary Eclipse fluorescence spectrophotometer was employed to acquire the fluorescence spectra of the CQDs. The morphology of the CQDs was captured by transmission electron microscopy (TEM) using a JEOL JEM-2100F instrument. X-ray photoelectron spectroscopy (XPS) was performed with the ESCALAB 220-Xi. Fourier transform infrared spectroscopy (FTIR, Nicolet iS50, Thermo Fisher, USA) was used to detect nanoparticle functional groups and chemical bonds. An HP inkjet printer (DeskJet 1112) was used for printing array patterns on filter paper. A smartphone (Huawei Mate 30) was utilized to read the fluorescence signals of the sensor arrays.

### Synthesis of CQDs

All CQDs were synthesized using a hydrothermal method, following previously published methods with slight modifications [35]. Briefly, 30 mg of ammonium dihydrogen citrate was mixed separately with 10 mg of polymyxin B, ampicillin, and gentamicin. The mixture was then ground into a powder and reacted in an oven at 180 °C for 4 h. The resulting black solid was dissolved in ultrapure water, followed by centrifugation at 10,000 rpm for 15 min to remove a significant amount of precipitate. Finally, the solution was subjected to dialysis using a dialysis membrane (MWCO 1000) to remove any unreacted molecules. The polymyxin CQDs (PM-CQDs), ampicillin CQDs (Amp-CQDs), and gentamicin CQDs (Gen-CQDs) were obtained, and the CQD solutions were stored at 4 °C for further experiments. The quantum yield (*Φ*) of the synthesized CQDs was determined by comparing their integrated photoluminescence intensities and absorbance values with those of quinine sulfate, a reference fluorophore with a known quantum yield (*Φ*_R_) of 0.54. The calculation followed the equation

$$\Phi = \left({\Phi }_{{\text{R}}} {S}_{1}*{A}_{1}*{\eta }_{1}^{2}\right)/ \left({S}_{2}*{A}_{2}*{\eta }_{2}^{2}\right)$$

where *S* represents the fluorescence peak area, *A* denotes absorbance, and *η* is the refractive index, set at 1.33 for both quinine sulfate in 0.1 M H_2_SO_4_ and the CQD solutions.

### Preparation and printing of inks

CQD fluorescence inks were prepared by mixing 5 mg/mL CQD, Triton X-100 as surfactant, and glycol as viscosity agent, in which the amounts of the latter two were optimized by checking the parameters of different ink formulations. Hydrophobic ink was prepared according to the literature [36]. AKD and Sudan red dyes were added to an n-heptane solution, where the Sudan red dye was helpful in visually distinguishing the sensor array from the hydrophobic region.

### Construction of the paper-based sensor array and visual detection device

First, hydrophobic ink and three fluorescent inks were injected into the black cartridge and three colorized cartridges of an inkjet printer, respectively. The printer was then used to print the sensor array pattern onto filter paper. Subsequently, the filter paper was baked in an oven at 100 °C for 5 min. For the visual detection device, a box measuring 10 cm × 10 cm × 10 cm was 3D-printed using black resin materials, with a sample slot of 3 cm × 5 cm. A 365-nm ultraviolet (UV) lamp and a UV filter were placed inside the box. The UV lamp irradiated the paper-based sensor array, while the UV filter reduced the UV signal reflected and scattered by the paper. The RGB value of the image was obtained using the smartphone software (Color Grab app).

### Bacterial preparation

*Escherichia coli* (*E. coli*, ATCC25312), *Pseudomonas aeruginosa* (*P. aeruginosa*, ATCC 15442), *Salmonella typhimurium* (*S. typhimurium*, ATCC14028), *Staphylococcus aureus* (*S. aureus*, ATCC 29213) and *Listeria monocytogenes* (*L. monocytogenes*, ATCC 19115) were used in this study. A single colony of the five types of bacteria grown in the solid Luria–Bertani (LB) medium was picked and washed twice with phosphate-buffered saline (PBS; 3 mL). Then the bacterial resuspension was measured with the BioSpectrometer to determine the OD value. After that, the bacterial resuspension was diluted to different concentrations (1.0 × 10^3^ colony-forming units (CFU)/mL, 1.0 × 10^4^ CFU/mL, 1.0 × 10^5^ CFU/mL, 1.0 × 10^6^ CFU/mL, 1.0 × 10^7^ CFU/mL) with PBS for use according to the standard curve of CFU–OD_600_ pre-measured.

### Bacterial identification

Firstly, different concentrations of bacteria were dropped onto the sensor array and incubated for 10 min. Subsequently, the paper-based sensor array was placed into the visual detection device, and the RGB value of the image was obtained using the Color Grab app. The relative signal intensity variety (ΔRGB) was calculated according to the following equation: ΔRGB = (R_0_ − R)/R_0_ + (G_0_ − G)/G_0_ + (B_0_ − B)/B_0_, where R_0_, G_0,_ B_0_ were the extracted red, green, and blue values of the image without the addition of bacteria, and R, G, and B were the extracted red, green, and blue values of the image with the addition of bacteria. To identify the five bacteria at the concentration of 1.0 × 10^3^ CFU/mL or others, the sensor array was carried out ten times to detect the bacteria, generating a 3 × 5 × 10 training matrix (3 CQDs × 5 bacteria × 10 repetitions). The data generated by the array were further processed using custom machine learning algorithms, including *k*-nearest neighbors (KNN), naive Bayes method, decision tree, linear discriminant analysis (LDA), and support vector machines (SVM).

## Results and discussion

### Characterization of functionalized CQDs and sensing mechanism

**Fig. 1:**
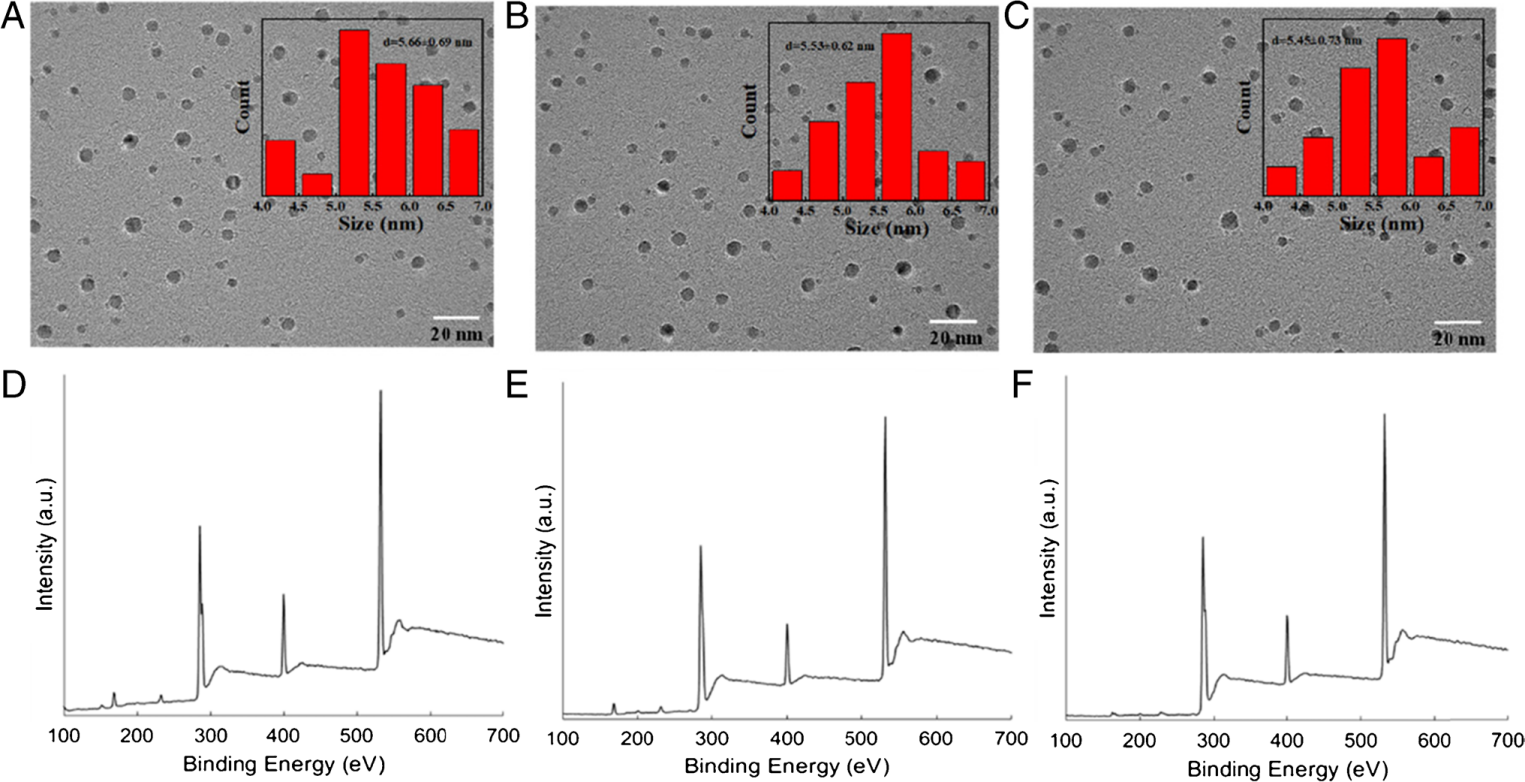
TEM image of (**A**) PM-CQDs, (**B**) Gen-CQDs, and (**C**) Amp-CQDs. Inset: diameter distribution of CQDs. XPS spectra of (**D**) PM-CQDs, (**E**) Gen-CQDs, and (**F**) Amp-CQDs

The three distinct functionalized CQDs, namely PM-CQDs, Gen-CQDs, and Amp-CQDs, were synthesized using a one-pot method. The TEM images (Fig. 1A–C) revealed that these CQDs have a uniform spherical morphology, with average sizes of 5.66 ± 0.69 nm, 5.53 ± 0.62 nm, and 5.45 ± 0.73 nm, respectively. XPS spectra, used to identify and characterize the chemical bonds and functional groups of the CQDs, revealed principal peaks for C 1*s* (285.03 eV), N 1*s* (400.02 eV), and O 1*s* (531.81 eV) (Fig. 1D–F). Moreover, the high-resolution XPS spectra (Figs. S1A–S3A) of the C 1*s* peak revealed three distinct sub-peaks attributed to the C=C/C–C bond (284.74 eV), C–N/C–O bond (285.72 eV), and C=N/C=O bond (288.02 eV) [37]. Additionally, the N 1*s* high-resolution spectrum (Figs. S1B–S3B) exhibited peaks at 398.81 eV and 399.98 eV, which can be categorized as O=C–N and C-N [38]. The O 1*s* spectrum (Figs. S1C–S3C) was fitted with C=O (530.95 eV) and C–O (532.32 eV) peaks [39]. A comprehensive analysis of the functional groups in CQDs was undertaken using FTIR. In Fig. S4, the absorption peak at 3443 cm^−1^ was attributed to the O–H group's stretching vibration. The peaks at 1706 cm^−1^ and 1624 cm^−1^ corresponded to the stretching vibrations of C=O and C=N, respectively. The peak at 1392 cm^−1^ aligns with the C=C structural feature. The presence of a C-O group was discerned from the peak at 1108 cm^−1^. These results unequivocally revealed that the CQDs are primarily composed of C, N, and O, with a notable abundance of carboxyl and hydroxyl groups on the surface.

**Fig. 2:**
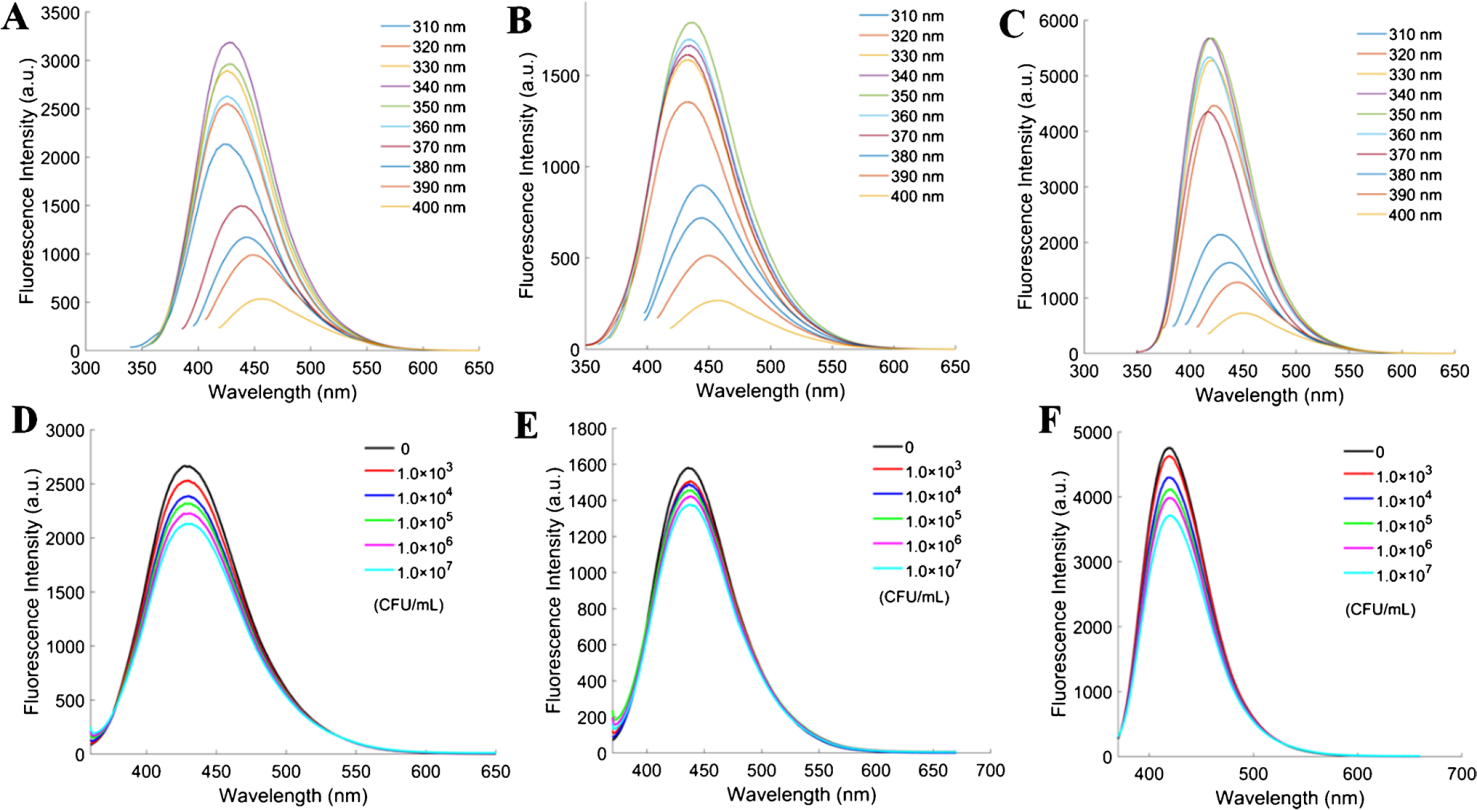
Fluorescence emission spectra of (**A**) PM-CQDs, (**B**) Gen-CQDs, and (**C**) Amp-CQDs in various excitation wavelengths. Fluorescence spectra of (**D**) PM-CQDs, (**E**) Gen-CQDs, and (**F**) Amp-CQDs after reaction with *E. coli* with different concentrations

The fluorescence emission spectra of PM-CQDs, Gen-CQDs, and Amp-CQDs across excitation wavelengths from 310 to 400 nm are presented in Fig. 2A–C. These results imply that the emission wavelength of the CQDs remains largely unaffected by variations in the excitation wavelength, while the fluorescence intensity is contingent upon it, with the optimal excitation wavelength falling within the range of 340 nm to 350 nm. The quantum yields of the three types of CQDs were calculated to be 10.7%, 13.2%, and 11.5%, respectively.

**Scheme 1:**
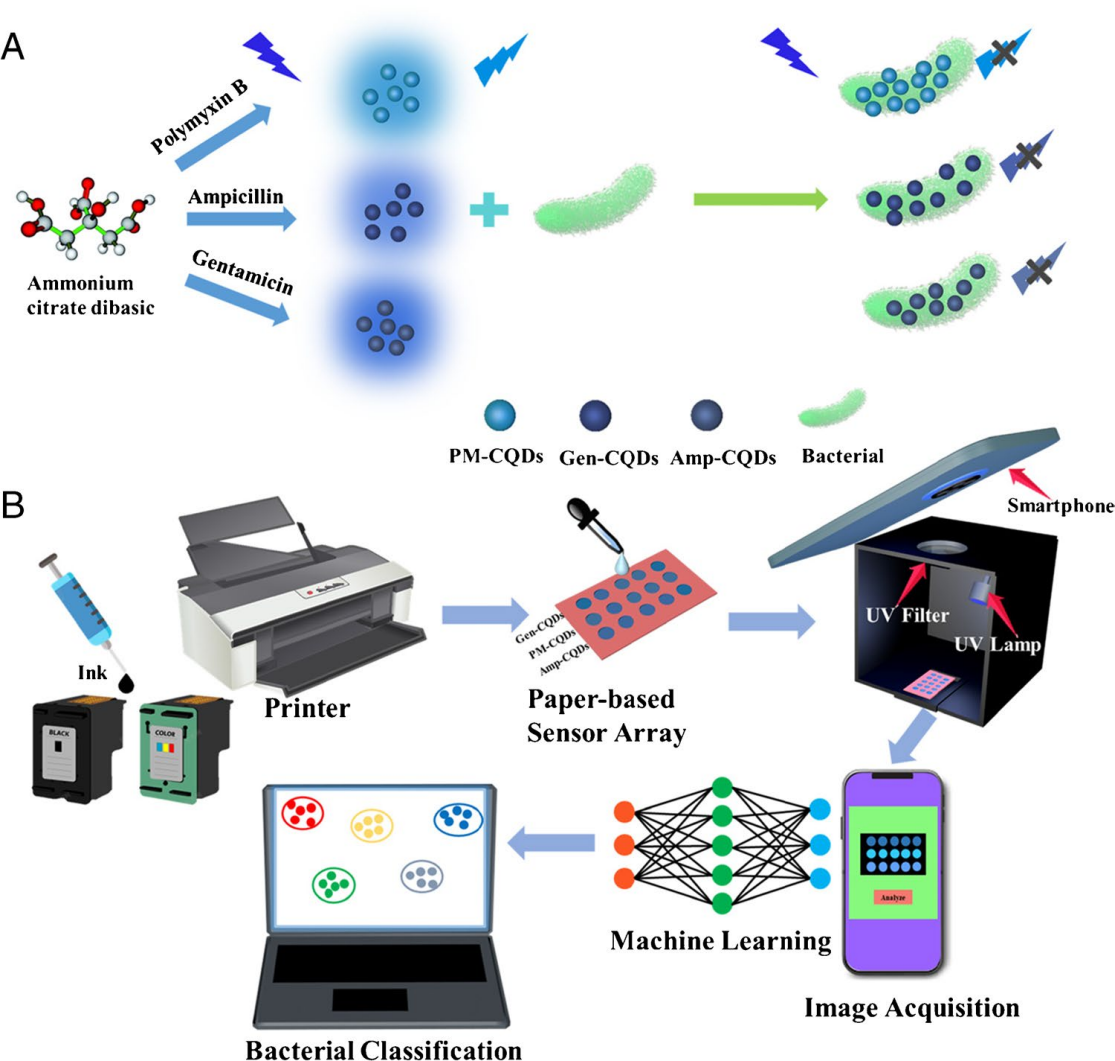
(**A**) Mechanism of bacterial detection based on CQDs. (**B**) Schematic of fabrication and bacterial recognition process of the fluorescence sensor-array platform

The notable reduction in fluorescence exhibited by the functionalized CQDs within the sensor array can be attributed to their aggregation on the surfaces of bacteria. These three CQDs exhibit varying affinities for bacteria, leading to distinct fluorescence quenching effects (Scheme 1A). These responses are utilized to create distinct bacterial recognition patterns. Consequently, the paper-based fluorescent sensor array developed in this study enables the straightforward identification of diverse bacterial strains.

### Fluorescence response of CQDs to bacteria

The feasibility of the proposed sensor array was assessed by examining the changes in fluorescence intensity of the three CQDs after exposure to *E. coli*. A clear correlation emerged between the concentration of *E. coli* and the reduction in fluorescence intensity of the CQDs, highlighting the capacity of bacteria to quench the fluorescence of these CQDs (Fig. 2D–F). Specifically, as the concentration of *E. coli* increased, the quenching efficiency of the CQDs progressively intensified. Remarkably, the highest quenching efficiency was observed when the concentration of *E. coli* was 1.0 × 10^7^ CFU/mL. Among the three types of CQDs, Amp-CQDs exhibited the highest quenching efficiency at an equivalent bacterial concentration.

As illustrated in Figs. S5—S8, the three CQDs also exhibited distinct fluorescence quenching patterns when incubated with four other bacterial strains. Consequently, sensor arrays comprising these three CQDs offer a valuable means of bacterial identification, as the diversity in the reactions of the CQDs with different bacterial strains facilitates discrimination.

### The construction of the platform

To enhance bacterial detection with a focus on speed, efficiency, and cost-effectiveness, we developed a novel sensor array platform. This platform integrates three key components: a paper-based sensor array for bacterial differentiation, a detection box for fluorescence excitation, and a smartphone for imaging. Scheme 1B illustrates this platform schematically, while Fig. S9 provides a visual representation. Notably, the paper-based sensor array can be printed on filter paper in just 10 s. The operational process involves dripping the solution onto the paper-based sensor array, placing the array into the detection box, and irradiating it with a UV lamp. Subsequently, the smartphone collects the signal through the port of the detection box.

The preparation of inks is a crucial step in the inkjet printing process, as the viscosity and surface tension of the inks directly impact the smooth ejection of ink droplets and their deposition on the filter paper surface. This, in turn, influences the sensing performance of the developed sensor array. To address this, we employed Triton X-100 and ethylene glycol to adjust the surface tension and viscosity of the ink, respectively. The optimal ratio was determined through a comparison with commercial inks. As displayed in Table 1, the addition amounts of Triton X-100 and ethylene glycol were set at 5% and 10%, respectively.

**Table 1:** Parameters of three fluorescence inks

Fluorescence inks	Particle size (nm)	Viscosity (mPa·s)	Surface tension (mN/m)
PM-CQDs	195	3.12	35.2
Gen-CQDs	212	3.22	33.9
Amp-CQDs	189	3.29	33.1
Commercial ink	370	2.86	36.2

### Identification of bacteria by sensor-array platform

To evaluate the capability of the fluorescence sensing array in identifying multiple bacterial strains, a diverse set of five bacteria was selected based on variations in size and morphology. These bacteria included *Pseudomonas aeruginosa*, *Escherichia coli*, *Staphylococcus aureus*, *Salmonella typhimurium*, and *Listeria monocytogenes*. Each bacterial strain, with a concentration ranging from 1.0 × 10^3^ CFU/mL to 1.0 × 10^7^ CFU/mL, was exposed to interaction with each of the three sensing units and subsequently assessed using the developed platform.

**Fig. 3:**
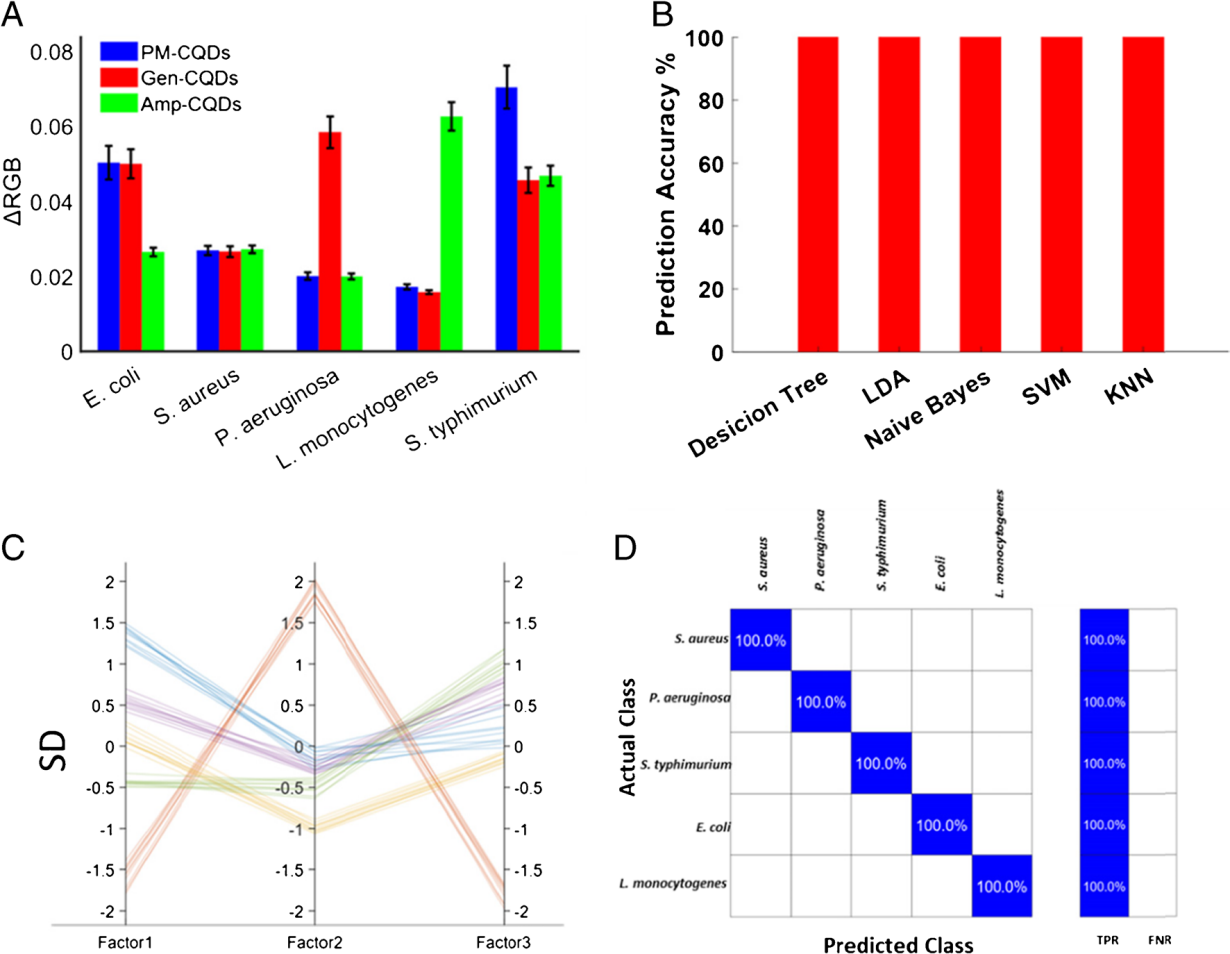
(**A**) Relative signal intensity variety (ΔRGB) of the three types of CQDs reacted with five types of bacteria in a concentration of 1.0 × 10^3^ CFU/mL. All values are the means of five replicates. (**B**) Identification efficiency of unknown individual samples. (**C**) Parallel coordinates figures of the three canonical factors (Factor 1, Factor 2, Factor 3) of unknown individual samples. (**D**) Confusion matrix plot of the results of the three classification algorithms for identification of individual unknown samples

Figure 3A illustrates the range of relative signal intensity variations (ΔRGB) observed in the three types of CQDs following their interaction with the five types of bacteria at a concentration of 1.0 × 10^3^ CFU/mL. Notably, the values of ΔRGB corresponding to the interactions between the different bacterial strains and the CQDs are distinctly unique. This can be attributed to the diverse binding abilities of the bacteria to the CQDs, which are determined by variations in size, morphology, and surface chemical components of the bacteria. Subsequently, the ΔRGB values were subjected to analysis using pattern recognition methods, including various machine learning algorithms.

During the training phase, we employed a random selection procedure to allocate 70% of the available data as the training set, while the remaining 30% was designated as the testing set. For each dataset, one or more ML algorithms were considered, depending on which algorithm demonstrated the best performance. We utilized all the available classification algorithms built into MATLAB for bacterial discrimination, without assuming that one algorithm was superior to the others. These algorithms included *k*-nearest neighbors (KNN), the naive Bayes method, decision trees, linear discriminant analysis (LDA), and support vector machines (SVM).

The test dataset consisted of a total of 122 samples, encompassing 50 individual bacterial samples, 24 binary mixtures, and 48 ternary mixtures derived from five distinct bacterial strains. This dataset was trained with several algorithms. Across the three subsets, the performance of the algorithms remained fairly consistent, with minor variations. The accuracy percentages of all these algorithms are illustrated in Figs. 3B and S10. Among these algorithms, three achieved a remarkable 100% accuracy rate on the entire dataset: (i) decision tree, (ii) linear discriminant analysis, (iii) naïve Bayes.

Three canonical factors, designated as Factor 1, Factor 2, and Factor 3, were generated for comprehensive discrimination. To gain a deeper understanding of the relationships among these three canonical factors derived from the ML algorithms, we employed a parallel coordinates figure (Figs. 3C and S11). A parallel coordinates figure offers a means of visualizing multivariate data and is particularly suitable for displaying trends in multivariate data. Additionally, the confusion matrix plot, displayed in Figs. 3D and S12, demonstrates that the three above-listed ML algorithms achieved 100% accuracy.

**Fig. 4:**
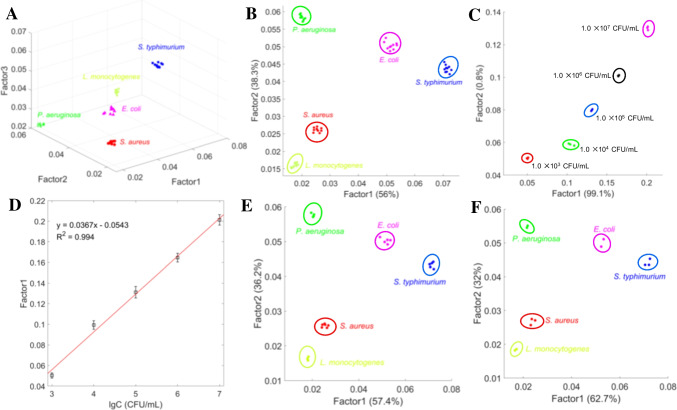
(**A**) Feature space plot of five types of bacteria using machine learning algorithms. (**B**) Canonical score plot for the identification of individual bacterial samples. The bacteria concentration was 1.0 × 10^3^ CFU/mL. (**C**) Canonical score plot for the distinction of *E. coli* at different concentrations. (**D**) Score plot of Factor 1 versus the concentration of *E. coli*. Error bars represent the standard deviation from three parallel tests. (**E**) Canonical score plot for the identification of unknown individual bacterial samples. (**F**) Canonical score plot for the identification of individual unknown bacterial samples in tap water. In each case, the bacteria concentration was 1.0 × 10^3^ CFU/mL

To further validate the discriminatory capabilities of the sensor array assisted by ML algorithms, we created a 3D feature space plot using the three canonical factors generated from a subset of individual bacterial samples (Fig. 4A). In this plot, each point represents the relative signal intensity variation of the three CQDs when reacted with a specific bacterial strain. The five bacterial strains were distinctly clustered, with a 100% identification accuracy rate for each type. We also selected the first two most significant discrimination factors to confirm the discrimination of the samples (Fig. 4B). Individual bacterial samples were identified without any failures, underscoring the high precision of the ML algorithms-assisted sensor array in bacterial discrimination. Furthermore, binary and ternary mixtures were accurately identified (Fig. S13). We calculated the Euclidean distances among the five types of bacteria to classify them.

To determine the limit of detection (LOD) of the sensor array, we conducted a quantitative analysis of five types of bacteria. The fluorescence response of the sensor array to varying concentrations of bacteria was transformed into canonical score plots. As shown in Fig. 4C, the sensor array effectively distinguishes between various concentrations of *E. coli*, correlating Factor 1 with bacterial concentration. Figure 4D demonstrates a strong linear relationship between the fluorescence intensity of the sensor array and the concentration of *E. coli*. The sensor array demonstrated consistent performance in distinguishing between the various concentrations of the remaining four bacteria (Fig. S14). Table S1 summarizes the LOD of the sensor array for the five types of bacteria. These results demonstrate that the sensor array can be utilized for simultaneous identification and quantification of bacteria.

### Identification of blind and real samples

The practicality of the platform was assessed by employing individual bacteria, binary mixtures, and ternary mixtures of the five bacterial strains, all with a total concentration of 1.0 × 10^3^ CFU/mL, as blind samples. As depicted in Figs. 4E and S15, a total of 45 blind samples were accurately identified (the results are listed in Table S2). The device's applicability to real samples was further explored using 12 unknown bacterial samples mixed in tap water, with each sample having a concentration of 1.0 × 10^3^ CFU/mL. All of the bacterial samples were successfully distinguished within the tap water medium (Fig. 4F, the results are listed in Table S3). These results confirm the platform's suitability for identifying the five types of bacteria in real samples.

## Conclusion

In this study, we developed a paper-based fluorescence sensor-array platform designed for the identification of various bacterial strains. Three antibiotic-modified CQDs, each with distinct bacterial binding affinities, were utilized as the sensing units. These CQDs were formulated into fluorescent inks with carefully adjusted surface tension and viscosity. The paper-based sensor array was then created by printing an array pattern directly onto filter paper using an inkjet printer.

The differential binding abilities of bacteria to the CQDs resulted in differences in the fluorescence quenching efficiency of the CQDs. Leveraging this principle, we established unique identification fingerprints for each bacterial strain on the proposed sensor-array platform. This platform enables rapid on-site discrimination of bacteria by utilizing smartphones and integrating multiple machine learning algorithms.

Furthermore, we validated the platform's applicability by successfully differentiating between bacterial strains in real samples and accurately identifying blind samples. This platform shows great promise as a tool for on-site testing, with significant potential applications in various fields such as food safety assessment, disease diagnosis, and environmental pollution detection.

### Supplementary Information

Below is the link to the electronic supplementary material.

Supplementary file1 (DOCX 3739 KB)

## Data Availability

The data that support the findings of this study are available from the corresponding author on request.
