# Supplementary material for: Paper-based fluorescence sensor array with functionalized carbon quantum dots for bacterial discrimination using a machine learning algorithm
Source: Anal Bioanal Chem. 2024 Apr 17;416(13):3139–48. doi: 10.1007/s00216-024-05262-4 (PMC11068836; doi:10.1007/s00216-024-05262-4)
Supplement: Supplementary file 1 — Supplementary file1 (DOCX 3739 KB) [file 216_2024_5262_MOESM1_ESM.docx]

**Supporting Information**

**Paper-based Fluorescence Sensor Array with Functionalized Carbon Quantum Dots for** **Bacterial Discrimination using a Machine Learning Algorithm**

Fangbin Wang^1^, Minghui Xiao^1^, Jing Qi^2, *^, Liang Zhu^3, **^

^1^School of Food and Biological Engineering, Hefei University of Technology, Hefei, 230009, China

^2^Department of Chemistry, National University of Singapore, Singapore, 117543, Singapore

^3^Department of Biomedical Engineering, The HongKong Polytechnic University, Hong Kong 999077, China

*Corresponding author: Jing Qi, QJ7@nus.edu.sg

**Corresponding author: Liang Zhu, liangzhu@polyu.edu.hk

**2.1. Description of Machine Learning Algorithms Used**

We have tried five types of ML algorithms to analyze our fluorescence data. A key feature of each ML algorithm has been described in the following paragraphs.

Nearest Neighbors (KNN) is a simple and intuitive machine learning algorithm used for classification and regression tasks. It works by finding the K training examples (data points with known labels) that are closest in distance to a new, unseen data point. The algorithm then predicts the label or value for the new data point based on the labels or values of its nearest neighbors. KNN is easy to understand and implement, making it a useful baseline algorithm for various machine learning tasks. However, its performance can be sensitive to the choice of the number of neighbors (K) and the distance metric used.

Naive Bayes is a probabilistic machine learning algorithm used for classification tasks. It calculates the probability of a data point belonging to a specific class based on the probability of its features. The "naive" assumption is that features are independent, simplifying calculations. It's commonly used in text classification, spam detection, and other tasks where feature independence approximation holds.

A Decision Tree is a machine learning algorithm used for both classification and regression tasks. It resembles a flowchart, where decisions are made at each node to split the data into subsets based on feature conditions. The goal is to reach a leaf node that provides the final prediction or value for the input data point. Decision Trees are interpretable and can handle both categorical and numerical data. They're often used in a variety of domains for their simplicity and transparency.

Linear Discriminant Analysis (LDA) is a dimensionality reduction and classification technique commonly used in machine learning and statistics. LDA seeks to find a linear combination of features that best separates multiple classes or groups in a dataset. It does this by maximizing the ratio of the between-class variance to the within-class variance. LDA is often employed in pattern recognition and classification tasks, especially when there are multiple classes and you want to reduce the dimensionality while preserving class-related information.

Support Vector Machines (SVM) is a powerful machine learning algorithm primarily used for classification tasks, but it can also be applied to regression. SVM works by finding the optimal hyperplane that best separates different classes of data points in a high-dimensional feature space. It aims to maximize the margin, which is the distance between the hyperplane and the nearest data points from each class. SVM is effective in handling both linearly separable and non-linearly separable data through the use of various kernel functions. It's known for its ability to handle high-dimensional data and its strong generalization performance.


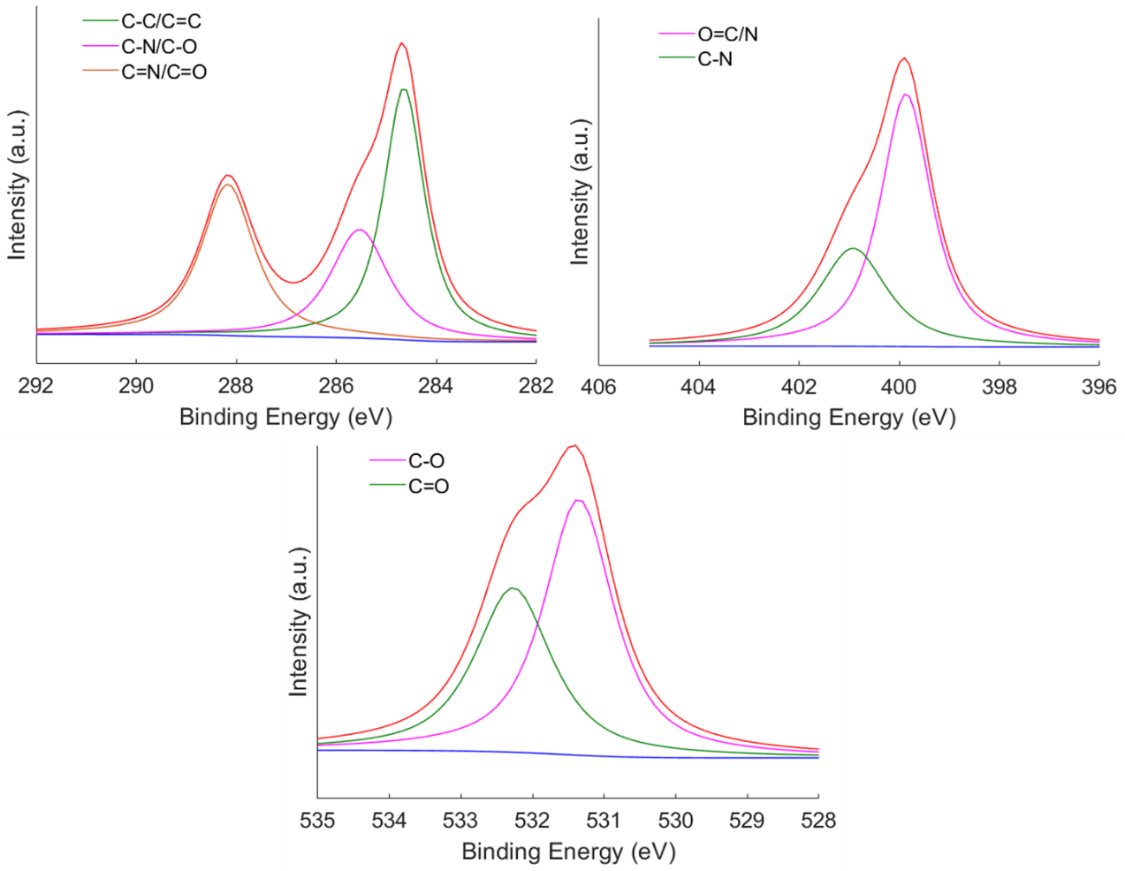


**Figure S1**. XPS spectra of PM-CQDs: (A) C 1s, (B) N 1s, and (C) O 1s.


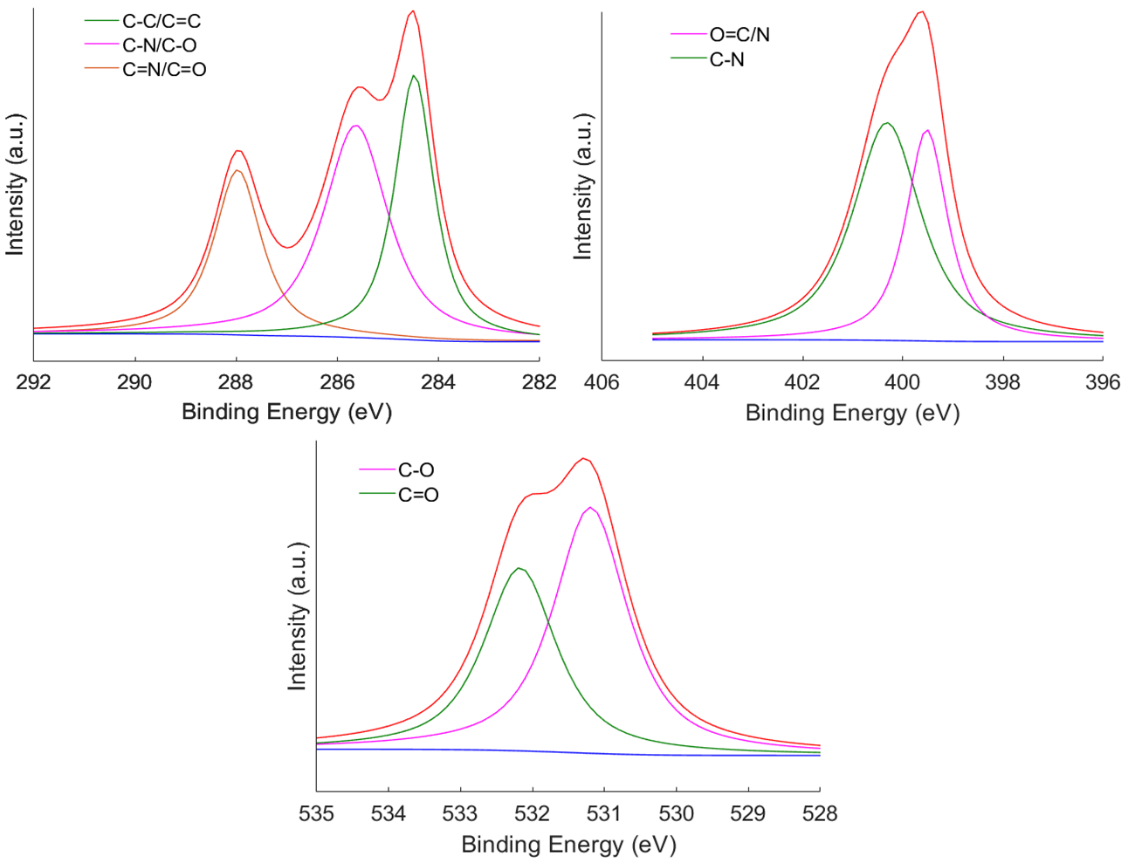


**Figure S2**. XPS spectra of Gen-CQDs: (A) C 1s, (B) N 1s, and (C) O 1s.


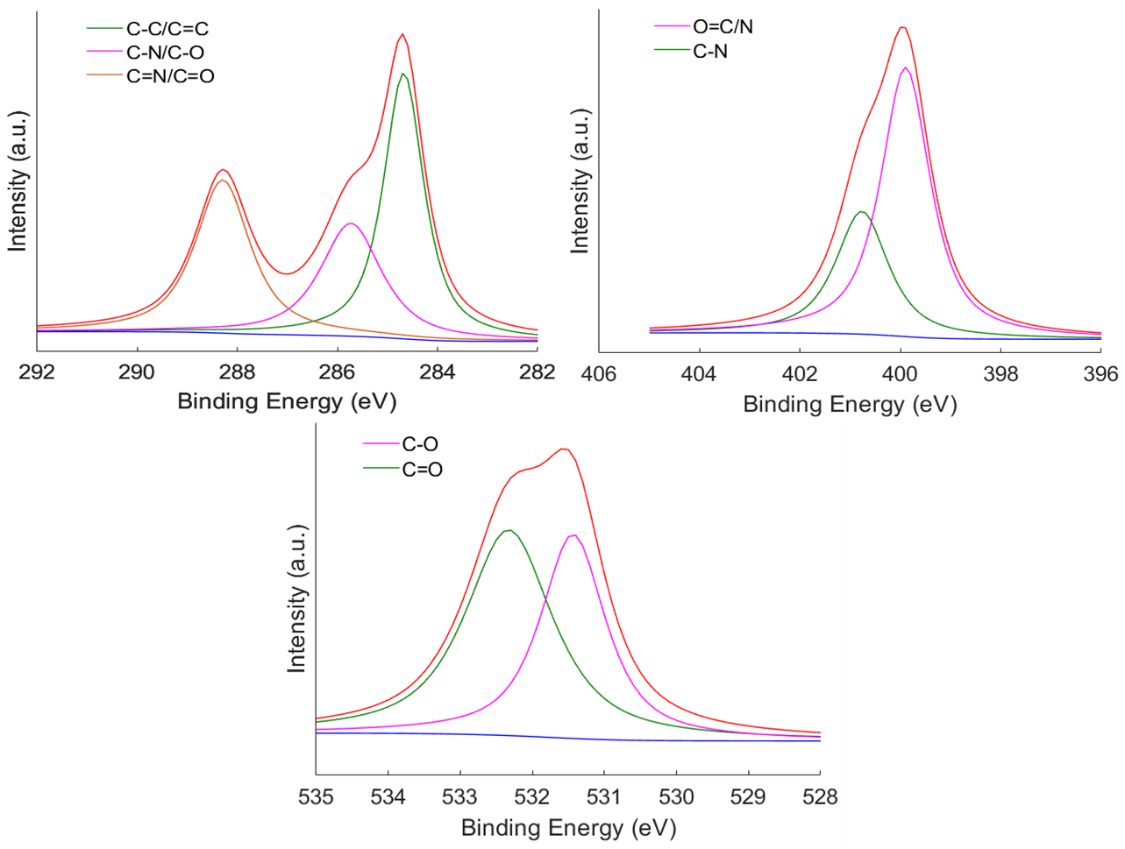


**Figure S3**. XPS spectra of Amp-CQDs: (A) C 1s, (B) N 1s, and (C) O 1s.


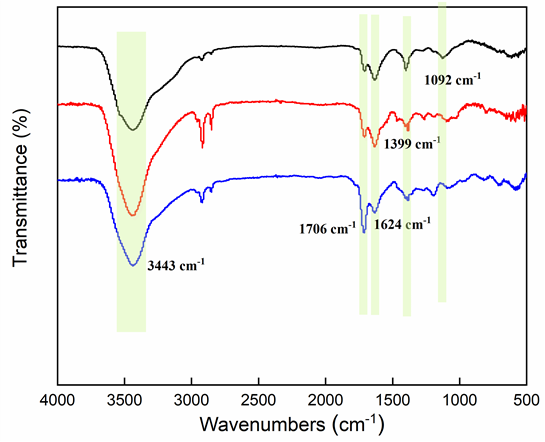


**Figure S4.** FT-IR of the PM-CQDs, Gen-CQDs and Amp-CQDs.


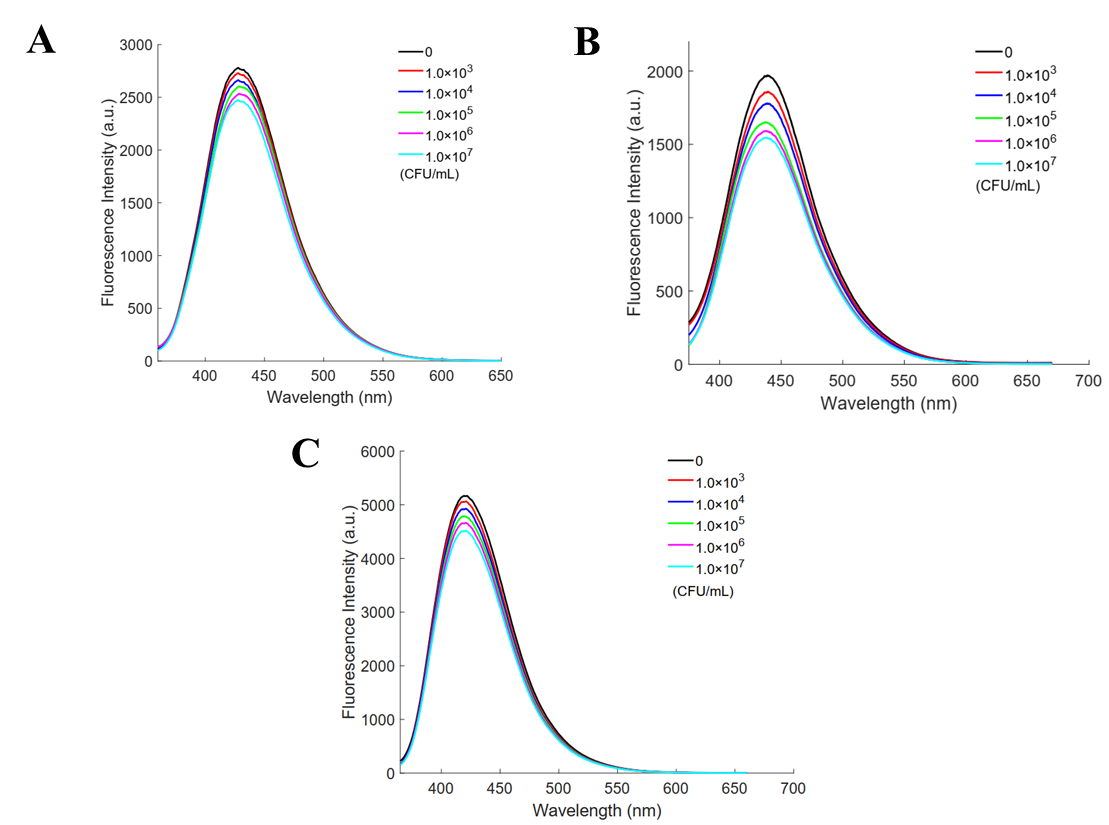


**Figure S5**. Fluorescence spectra of (A) PM-CQDs, (B) Gen-CQDs and (C) Amp-CQDs after reaction with *P. aeruginosa* with different concentrations.


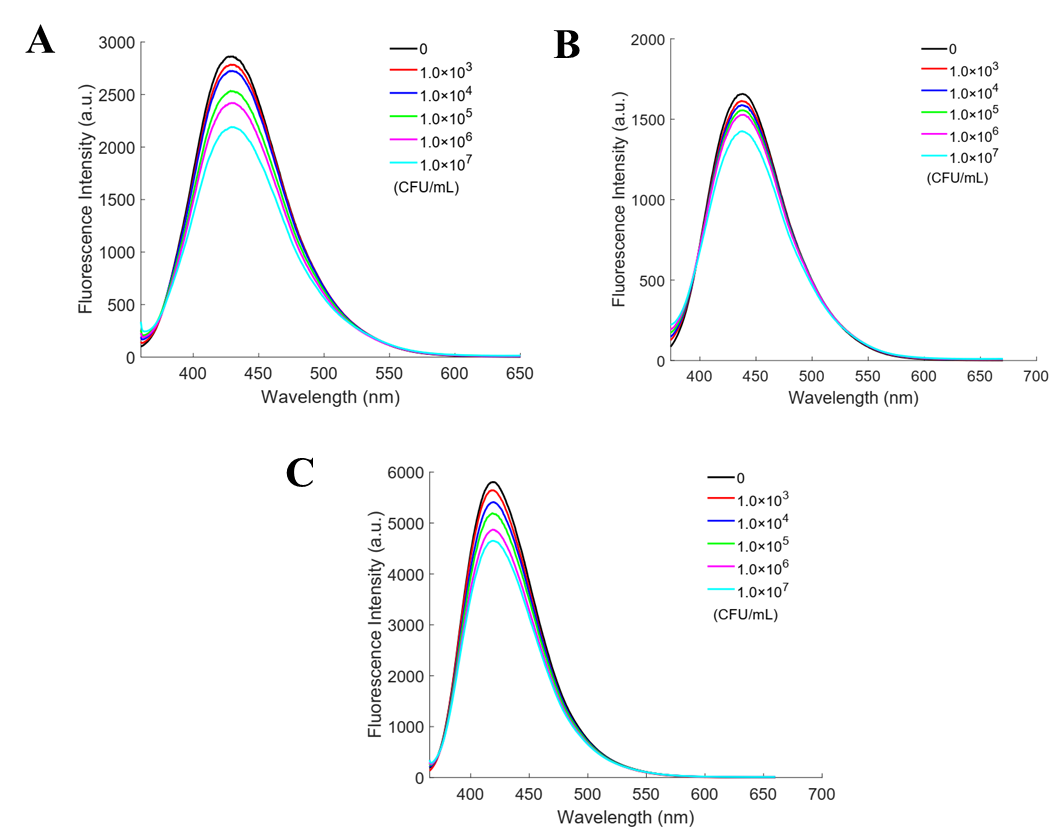


**Figure S6**. Fluorescence spectra of (A) PM-CQDs, (B) Gen-CQDs and (C) Amp-CQDs after reaction with *S. aureus* with different concentrations.


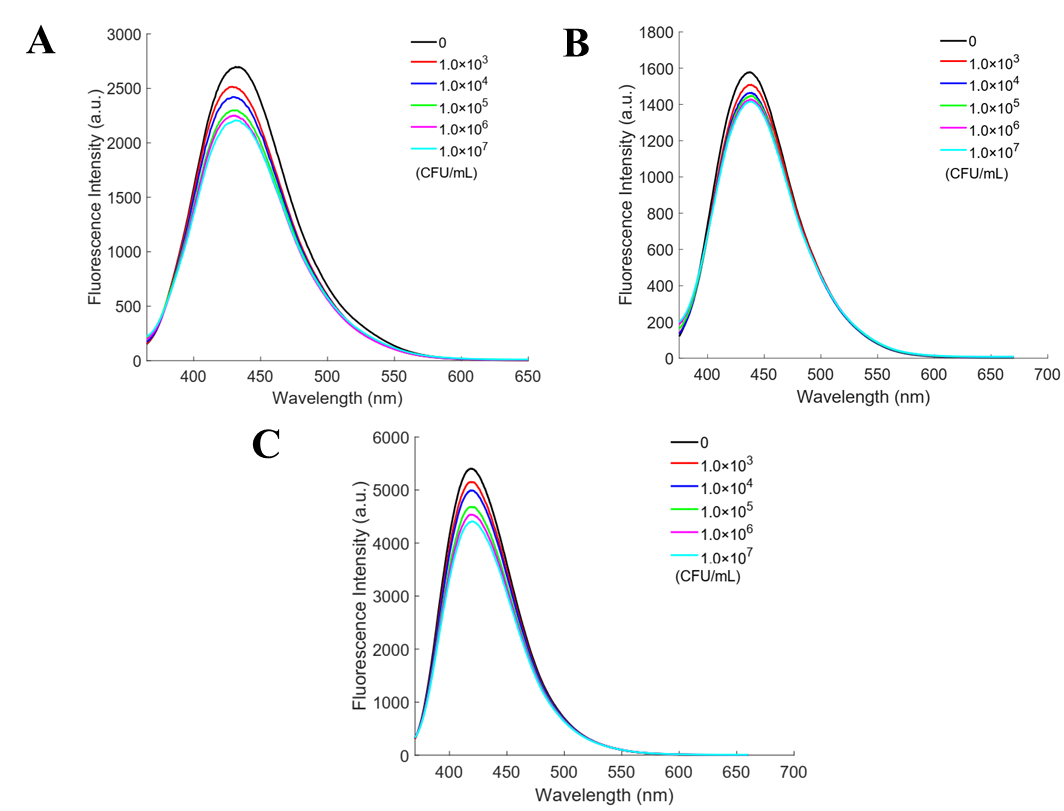


**Figure S7**. Fluorescence spectra of (A) PM-CQDs, (B) Gen-CQDs and (C) Amp-CQDs after reaction with *S. typhimurium* with different concentrations.


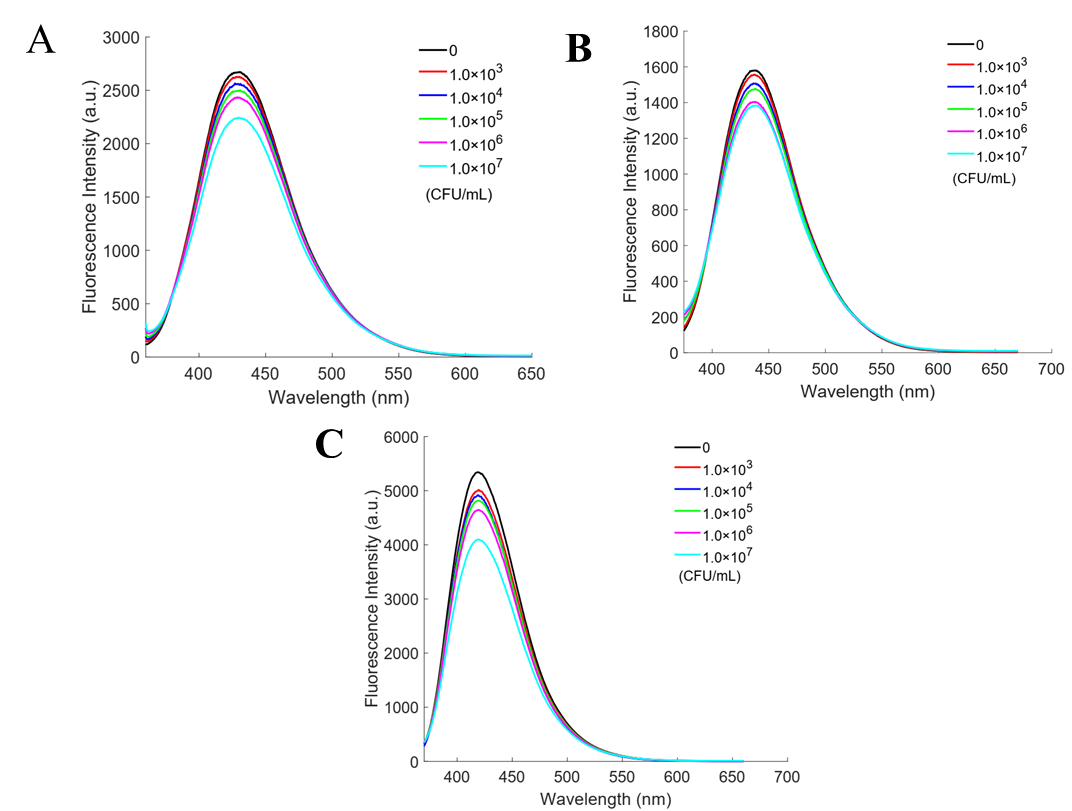


**Figure S8**. Fluorescence spectra of (A) PM-CQDs, (B) Gen-CQDs and (C) Amp-CQDs after reaction with *L. monocytogenes* with different concentrations.


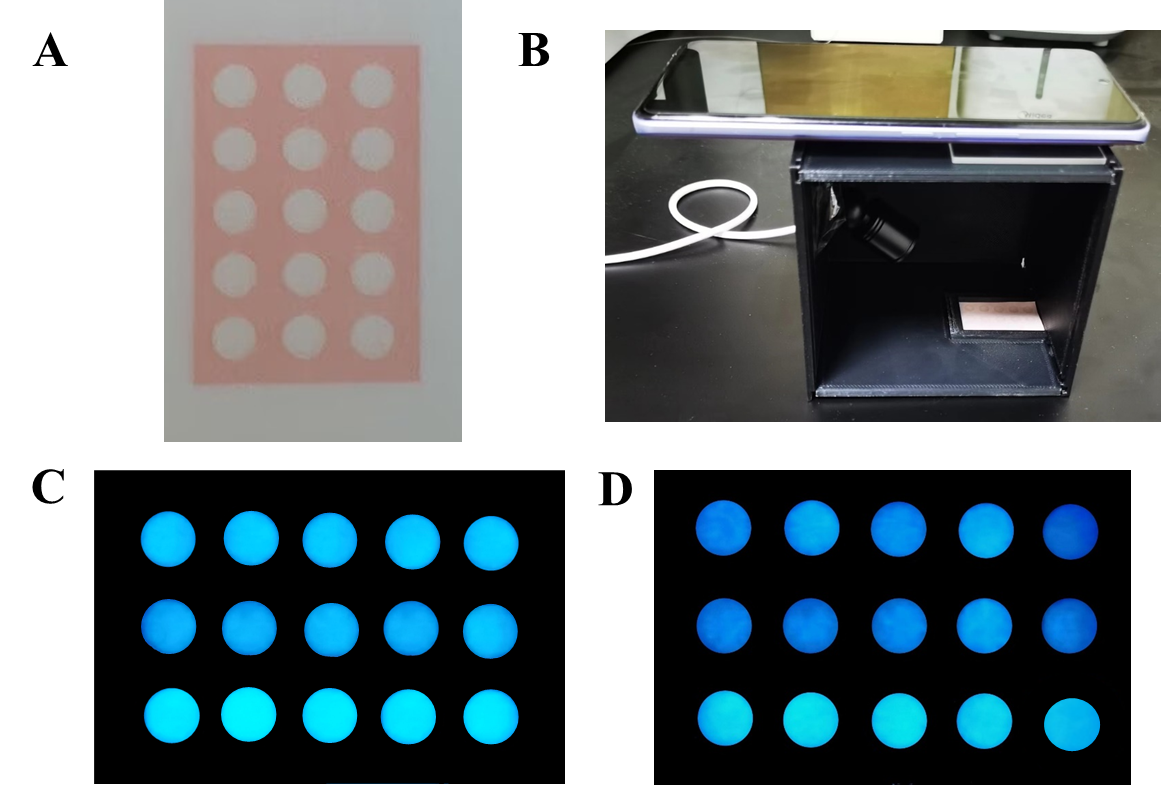


**Figure S9**. (A) Photos of paper-based sensor array. (B) Photo of the detection platform. (C) Photos of paper-based sensor array without bacteria, (D) with bacteria under ultraviolet lamp.

**Figure S10.** Identification efficiency of unknown (A) binary mixtures and (B) ternary mixtures from five kinds of bacteria with the concentration of 1.0 🞨 10^3^ CFU/mL using machine learning methods for pattern recognition.


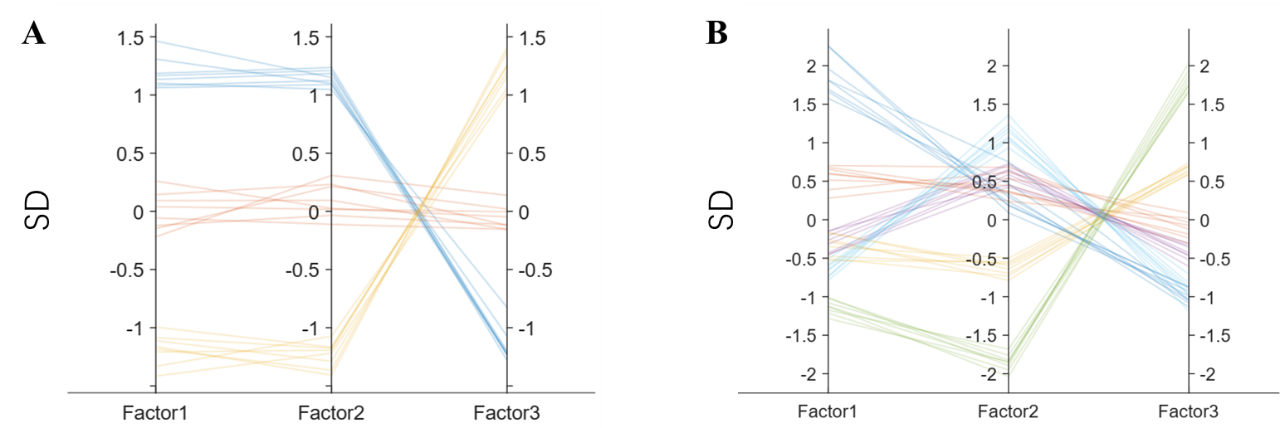


**Figure S11.** Parallel coordinates figures of the three canonical factors (Factor1, Factor2, Factor3) of unknown (A) binary mixtures and (B) ternary mixtures from five kinds of bacteria with the concentration of 1.0 🞨 10^3^ CFU/mL.


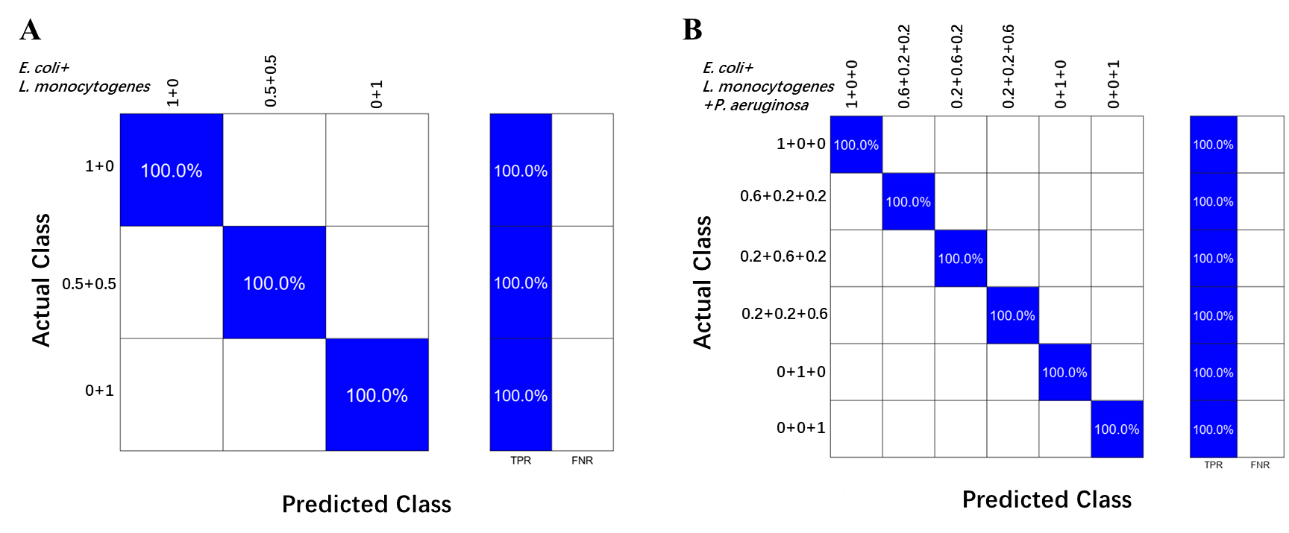


**Figure S12.** Confusion matrix plot of the output of the classifier from the three algorithms for detection of (A) binary mixtures and (B) ternary mixtures from five kinds of bacteria with the concentration of 1.0 🞨 10^3^ CFU/mL.


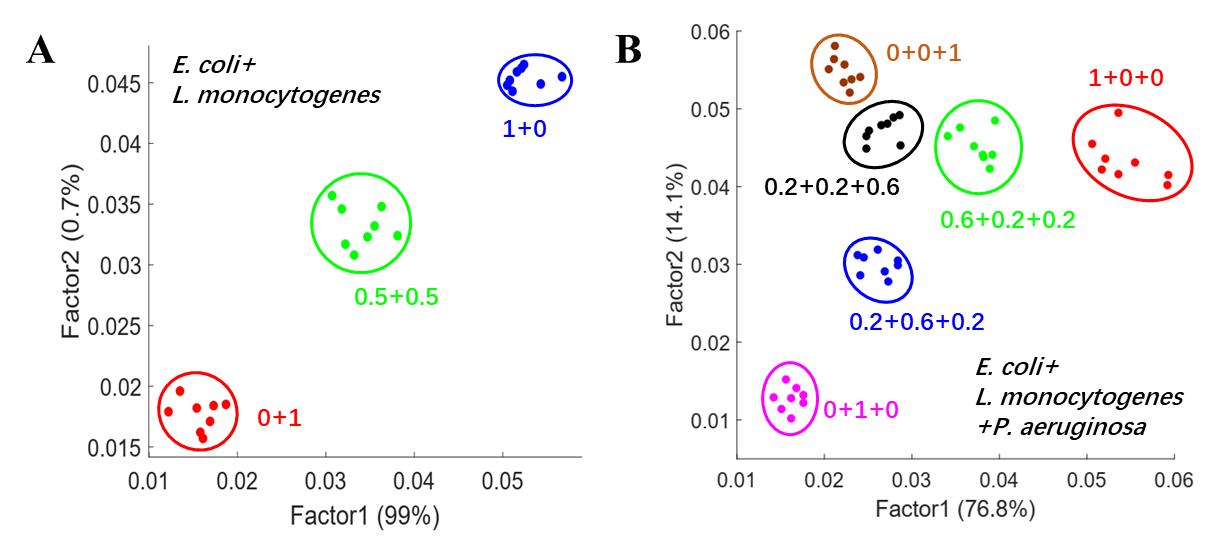


**Figure S13.** Canonical score plot for the discrimination of (A) binary mixtures (*E. coli* and *L. monocytogenes* with different ratios), and (B) ternary mixtures (*E. coli*, *P. aeruginosa* and *L. monocytogenes* with different ratios). In each case, the bacteria concentration was 1.0 🞨 10^3^ CFU/mL.

**Figure S14.** canonical score plot for the distinction of (A) *P. aeruginosa*, (C) *S. aureus* and (E) *S. typhimurium* (G) *L. monocytogenes* at different concentrations. Score plot of factor1 versus the concentration of (B) *P. aeruginosa,* (D) *S. aureus and* (F) *S. typhimurium* (H) *L. monocytogenes*. Error bars represent the standard deviation from three parallel tests.

**
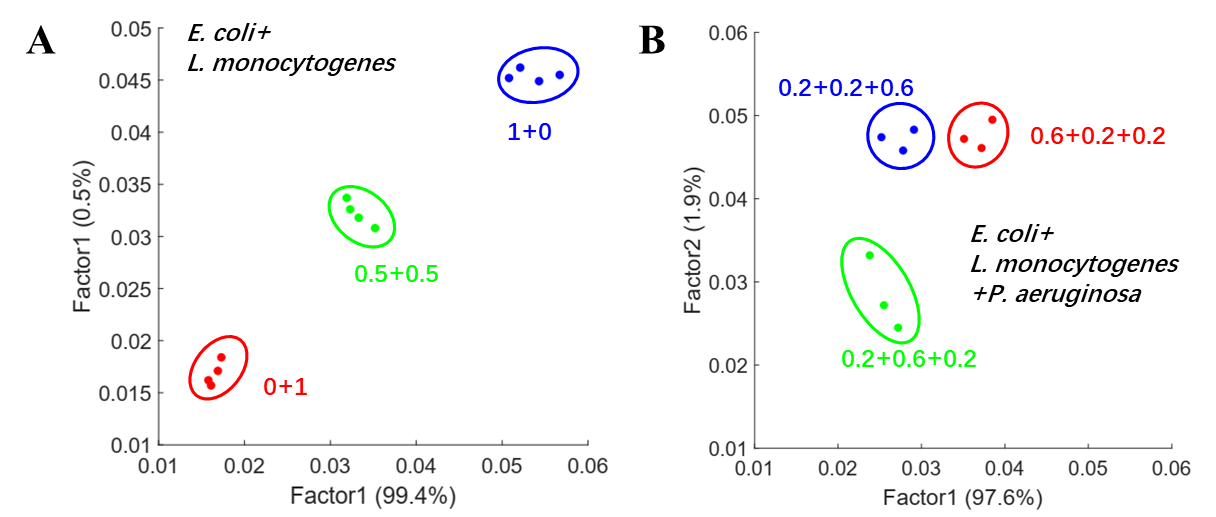
**

**Figure S15.** Canonical score plot for the discrimination of unknown (A) binary mixtures (*E. coli* and *L. monocytogenes* with different ratios), and (B) ternary mixtures (*E. coli*, *P. aeruginosa* and *L. monocytogenes* with different ratios) with the concentration of 1.0 🞨 10^3^ CFU/mL.

**Table S1.** Formulas for quantitative detection of bacteria by Factor1 scores

| **Bacteria** | **Equations** | ***R*^2^** | **Range (CFU/mL)** | **LOD (CFU/mL)** |
| --- | --- | --- | --- | --- |
| *E. coli*. | y=0.0367x-0.0543 | 0.994 | 10^3^-10^7^ | 277 |
| *P. aeruginosa* | y=0.0228x-0.0464 | 0.996 | 10^3^-10^7^ | 446 |
| *S. aureus* | y=0.0456x-0.1179 | 0.993 | 10^3^-10^7^ | 223 |
| *S. typhimurium* | y=0.0285x-0.0114 | 0.989 | 10^3^-10^7^ | 357 |
| *L. monocytogenes* | y=0.0261x-0.0609 | 0.991 | 10^3^-10^7^ | 390 |

**Table S2.** Identification of 24 unknown individual bacteria, 12 unknown binary mixtures and 9 unknown ternary mixtures. In each case, a bacteria concentration of 1.0 🞨 10^3^ CFU/mL was used.

| **NO.** | **Relative Fluorescence Change** | | | **Predicted** | **Actual** |
| --- | --- | --- | --- | --- | --- |
|  | **PM-CQDs** | **Gen-CQDs** | **Amp-CQDs** |  |  |
| 1 | 0.0253 | 0.0264 | 0.0277 | *S. aureus* | *S. aureus* |
| 2 | 0.0203 | 0.0571 | 0.0224 | *P. aeruginosa* | *P. aeruginosa* |
| 3 | 0.0524 | 0.0492 | 0.0245 | *E. coli* | *E. coli* |
| 4 | 0.0708 | 0.0427 | 0.0462 | *S. typhimurium* | *S. typhimurium* |
| 5 | 0.0179 | 0.0163 | 0.0607 | *L. monocytogenes* | *L. monocytogenes* |
| 6 | 0.0536 | 0.0504 | 0.0236 | *E. coli* | *E. coli* |
| 7 | 0.0721 | 0.0442 | 0.0475 | *S. typhimurium* | *S. typhimurium* |
| 8 | 0.0255 | 0.0261 | 0.0264 | *S. aureus* | *S. aureus* |
| 9 | 0.0183 | 0.0169 | 0.0616 | *L. monocytogenes* | *L. monocytogenes* |
| 10 | 0.0205 | 0.0573 | 0.0219 | *P. aeruginosa* | *P. aeruginosa* |
| 11 | 0.0243 | 0.0261 | 0.0273 | *S. aureus* | *S. aureus* |
| 12 | 0.0523 | 0.0505 | 0.0251 | *E. coli* | *E. coli* |
| 13 | 0.0705 | 0.0435 | 0.0466 | *S. typhimurium* | *S. typhimurium* |
| 14 | 0.0195 | 0.0583 | 0.0225 | *P. aeruginosa* | *P. aeruginosa* |
| 15 | 0.0179 | 0.0163 | 0.0607 | *L. monocytogenes* | *L. monocytogenes* |
| 16 | 0.0241 | 0.0253 | 0.0281 | *S. aureus* | *S. aureus* |
| 17 | 0.0511 | 0.0499 | 0.0254 | *E. coli* | *E. coli* |
| 18 | 0.0715 | 0.0438 | 0.0452 | *P. aeruginosa* | *P. aeruginosa* |
| 19 | 0.0181 | 0.0158 | 0.0613 | *L. monocytogenes* | *L. monocytogenes* |
| 20 | 0.0723 | 0.0421 | 0.0474 | *S. typhimurium* | *S. typhimurium* |
| 21 | 0.0491 | 0.0513 | 0.0235 | *E. coli* | *E. coli* |
| 22 | 0.0269 | 0.0259 | 0.0258 | *S. typhimurium* | *S. typhimurium* |
| 23 | 0.0263 | 0.0252 | 0.0282 | *S. aureus* | *S. aureus* |
| 24 | 0.0207 | 0.0578 | 0.0217 | *P. aeruginosa* | *P. aeruginosa* |
| 25 | 0.0521 | 0.0462 | 0.0205 | 100% *E. coli* +0% *L. monocytogenes* | 100% *E. coli* +0% *L. monocytogenes* |
| 26 | 0.0543 | 0.0449 | 0.0242 | 100% *E. coli* +0% *L. monocytogenes* | 100% *E. coli* +0% *L. monocytogenes* |
| 27 | 0.0567 | 0.0455 | 0.0214 | 100% *E. coli* +0% *L. monocytogenes* | 100% *E. coli* +0% *L. monocytogenes* |
| 28 | 0.0508 | 0.0452 | 0.0217 | 100% *E. coli* +0% *L. monocytogenes* | 100% *E. coli* +0% *L. monocytogenes* |
| 29 | 0.0323 | 0.0326 | 0.0457 | 50% *E. coli* +50% *L. monocytogenes* | 50% *E. coli* +50% *L. monocytogenes* |
| 30 | 0.0319 | 0.0337 | 0.0438 | 50% *E. coli* +50% *L. monocytogenes* | 50% *E. coli* +50% *L. monocytogenes* |
| 31 | 0.0333 | 0.0318 | 0.0422 | 50% *E. coli* +50% *L. monocytogenes* | 50% *E. coli* +50% *L. monocytogenes* |
| 32 | 0.0352 | 0.0308 | 0.0436 | 50% *E. coli* +50% *L. monocytogenes* | 50% *E. coli* +50% *L. monocytogenes* |
| 33 | 0.0161 | 0.0157 | 0.0685 | 0% *E. coli* +100% *L. monocytogenes* | 0% *E. coli* +100% *L. monocytogenes* |
| 34 | 0.0158 | 0.0162 | 0.0678 | 0% *E. coli* +100% *L. monocytogenes* | 0% *E. coli* +100% *L. monocytogenes* |
| 35 | 0.0169 | 0.0171 | 0.0641 | 0% *E. coli* +100% *L. monocytogenes* | 0% *E. coli* +100% *L. monocytogenes* |
| 36 | 0.0173 | 0.0184 | 0.0657 | 0% *E. coli* +100% *L. monocytogenes* | 0% *E. coli* +100% *L. monocytogenes* |
| 37 | 0.0385 | 0.0495 | 0.0361 | 60% *E. coli* +20% *L. monocytogenes* +20% *P. aeruginosa* | 60% *E. coli* +20% *L. monocytogenes* +20% *P. aeruginosa* |
| 38 | 0.0372 | 0.0461 | 0.0372 | 60% *E. coli* +20% *L. monocytogenes* +20% *P. aeruginosa* | 60% *E. coli* +20% *L. monocytogenes* +20% *P. aeruginosa* |
| 39 | 0.0351 | 0.0472 | 0.0355 | 60% *E. coli* +20% *L. monocytogenes* +20% *P. aeruginosa* | 60% *E. coli* +20% *L. monocytogenes* +20% *P. aeruginosa* |
| 40 | 0.0272 | 0.0245 | 0.2045 | 20% *E. coli* +60% *L. monocytogenes* +20% *P. aeruginosa* | 20% *E. coli* +60% *L. monocytogenes* +20% *P. aeruginosa* |
| 41 | 0.0255 | 0.0272 | 0.0468 | 20% *E. coli* +60% *L. monocytogenes* +20% *P. aeruginosa* | 20% *E. coli* +60% *L. monocytogenes* +20% *P. aeruginosa* |
| 42 | 0.0238 | 0.0332 | 0.0486 | 20% *E. coli* +60% *L. monocytogenes* +20% *P. aeruginosa* | 20% *E. coli* +60% *L. monocytogenes* +20% *P. aeruginosa* |
| 43 | 0.0291 | 0.0483 | 0.0313 | 20% *E. coli* +20% *L. monocytogenes* +60% *P. aeruginosa* | 20% *E. coli* +20% *L. monocytogenes* +60% *P. aeruginosa* |
| 44 | 0.0252 | 0.0474 | 0.0321 | 20% *E. coli* +20% *L. monocytogenes* +60% *P. aeruginosa* | 20% *E. coli* +20% *L. monocytogenes* +60% *P. aeruginosa* |
| 45 | 0.0278 | 0.0458 | 0.0301 | 20% *E. coli* +20% *L. monocytogenes* +60% *P. aeruginosa* | 20% *E. coli* +20% *L. monocytogenes* +60% *P. aeruginosa* |

**Table S3** Identification of 12 unknown individual bacteria samples in tap water. In each case, a bacteria concentration of 1.0 🞨 10^3^ CFU/mL was used.

| **NO.** | **Relative Fluorescence Change** | | | **Predicted** | **Actual** |
| --- | --- | --- | --- | --- | --- |
|  | **PM-CQDs** | **Gen-CQDs** | **Amp-CQDs** |  |  |
| 1 | 0.0725 | 0.0453 | 0.0469 | *S. typhimurium* | *S. typhimurium* |
| 2 | 0.0215 | 0.0545 | 0.0236 | *P. aeruginosa* | *P. aeruginosa* |
| 3 | 0.0249 | 0.0271 | 0.0282 | *S. aureus* | *S. aureus* |
| 4 | 0.0172 | 0.0186 | 0.0611 | *L. monocytogenes* | *L. monocytogenes* |
| 5 | 0.0721 | 0.0435 | 0.0482 | *S. typhimurium* | *S. typhimurium* |
| 6 | 0.0533 | 0.0511 | 0.0262 | *E. coli* | *E. coli* |
| 7 | 0.0165 | 0.0182 | 0.0613 | *L. monocytogenes* | *L. monocytogenes* |
| 8 | 0.0231 | 0.0257 | 0.0266 | *S. aureus* | *S. aureus* |
| 9 | 0.0705 | 0.0435 | 0.0482 | *S. typhimurium* | *S. typhimurium* |
| 10 | 0.0218 | 0.0551 | 0.0231 | *P. aeruginosa* | *P. aeruginosa* |
| 11 | 0.0235 | 0.0275 | 0.0281 | *S. aureus* | *S. aureus* |
| 12 | 0.0523 | 0.0489 | 0.0236 | *E. coli* | *E. coli* |
